# Perioperative analgesia after intrathecal morphine or local infiltration anesthesia for total knee replacement: A protocol for randomized controlled trial

**DOI:** 10.1097/MD.0000000000022394

**Published:** 2020-09-25

**Authors:** Zhengrong Qi, Ai Guo, Lifeng Ma, Zhiyao Li, Bo Yang, Jingxin Zhang

**Affiliations:** Department of Orthopedics, Beijing Friendship Hospital, Capital Medical University, Beijing, China.

**Keywords:** intrathecal morphine, local infiltration anesthesia, protocol, total knee replacement

## Abstract

**Objective:**

We perform this protocol for randomized controlled trial to compare the efficacy of intrathecal morphine and local infiltration anesthesia (LIA) in the treatment of the postoperative pain after total knee replacement (TKR).

**Methods:**

This is a randomized controlled, single center trial which was performed from March 2019 to March 2020. This trial is conducted according to the SPIRIT Checklist of randomized researches. It is authorized via the Ethics Committee of Beijing Friendship Hospital (2019-P2-050-01). Eighty participants who undergo TKR were randomized into 2 groups. Intrathecal morphine group: 0.1 mg of the morphine was intrathecally injected, and the spinal anesthetic was injected at the same time in the group LIA; In the LIA group: the knee joint was infiltrated with epinephrine, ketorologic acid and ropivacaine in the process of operation, and the identical mixture was injected 2 bolus through the intraarticular catheter after operation. The main outcome variables were the visual analog scale and the consumption amount of opioid every 6-hour interval within 2 days postoperatively. The secondary outcome variables were the side effects associated with opioid, the length of hospital stay, motion range, and the loss of blood collected by the closed suction drainage. All the required analyses were carried out via applying the SPSS for Windows Version 19.0.

**Results:**

The clinical outcome variables between groups were shown in Table 1.

**Conclusion:**

This protocol will provide the evidence on which technique can achieve better analgesia after TKR.

## 1 Introduction

Total knee replacement (TKR) is a successful surgical approach for the treatment of end-stage knee osteoarthritis in terms of functional recovery and pain relief.^[1,2]^ The demand for primary TKR is estimated to grow by 3.5 million procedures in the United States by 2030.^[3]^ Although TKR is popular, approximately 20% of the patients are not satisfied. Published studies indicated that an estimated 40% to 60% of patients report severe pain following TKR, which is the common reasons for dissatisfaction.^[4,5]^ The aggravation of pain is associated with the proinflammatory states, pain regulation system damage and the tissue damage. Inadequate perioperative analgesia may be related to the increased costs, prolonged hospital stay and adverse clinical outcomes.^[6,7]^ Many methods have been applied to reduce pain following TKR, including local infiltration analgesia (LIA), oral analgesics, peripheral nerve block, and intrathecal morphine.^[8–11]^ However, the analgesic efficacy of these techniques and their effect on postoperative opioid consumption remains unclear.

LIA via an infusion catheter has been shown to be more effective than single bolus administration, is easy to perform and delivers analgesia directly to the source of pain. Wound infection is a major concern, which is disastrous for arthroplasties. Intrathecal morphine may be delivered at the same time as spinal anesthesia and therefore the additional time required is negligible. One disadvantage, however, is the potential risk of opiate toxicity. In addition, opioid can lead to many side effects, for instance, respiratory depression, gastrointestinal reactions, constipation and urine retention.^[12,13]^ Therefore, multimodal analgesia has been extensively used in perioperative period of TKR. Currently, perioperative analgesia after intrathecal morphine or LIA for TKR still controversial. We perform this protocol for randomized controlled trial to compare the efficacy of intrathecal morphine and LIA in the treatment of the postoperative pain after TKR.

## 2 Methods

### 2.1 Study design

This is a randomized controlled, single center trial which was implemented from March 2019 to March 2020. This trial is conducted according to the SPIRIT Checklist of randomized researches. It was authorized via the Ethics Committee of Beijing Friendship Hospital (2019-P2-050-01), and it has been registered in the research registry (researchregistry5942).

### 2.2 Patients

Eighty participants who undergo TKR were analyzed. In the random envelope, all patients were assigned a random number via using the random number Table 1, and the result of allocation was hidden. Patients were randomly divided into LIA group (with 40 patients) and the intrathecal morphine (with 40 patients). Inclusion criteria contains

1. elective primary unilateral TKR;
2. people between the ages of 50 and 70;
3. BMI less than 40 kg/m^2^;
4. the acceptance of patients to participate in this work.

**Table 1:**
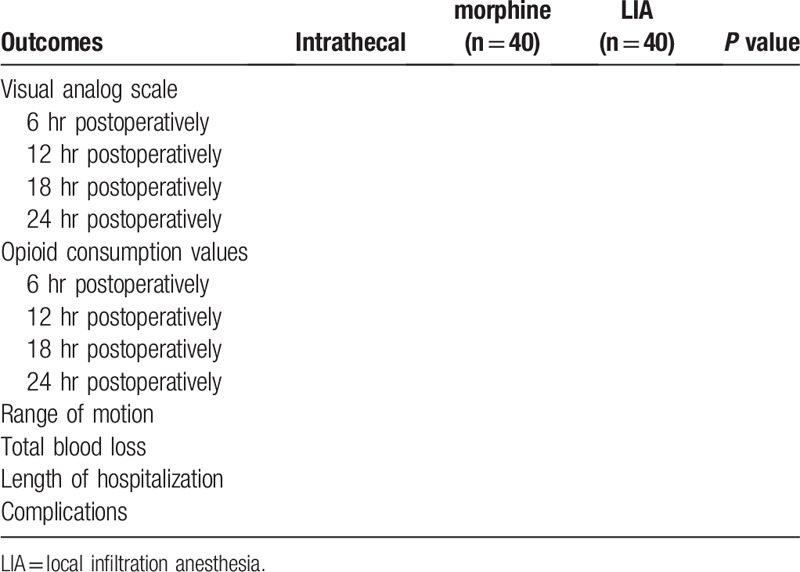
Outcome measures between intrathecal morphine and local infiltration anesthesia after total knee replacement.

The exclusion criteria contains:

1. patients with the history of severe renal and hepatic dysfunction;
2. contraindication to spinal anesthesia (refusal, coagulopathy, sepsis, local infection, spinal defects, previous laminectomy);
3. Contraindication to nonsteroidal antiinflammatory drugs;
4. Patient has an emotional or neurological condition that would prevent their willingness to participate in the study.

### 2.3 Analgesic protocol

All the patients were given 10 mg of diazepam orally 1 hour before the planned operation, and all the surgeries were implemented under the spinal anesthesia, and a 27-G pencil-point spinal needle was utilized in the intervertebral space of L3/L4, with patient in sitting position. In the morphine group, 0.1 mg of the morphine (0.25 ml) was intrathecally injected, and the same volume of 0.9% normal saline and 17.5 mg of bupivacaine without glucose were injected into the LIA group. All the patients were given propofol intravenously or continued intraoperative infusion as required. If the patient experienced pain in the process of operation, the dosage of fentanyl intravenous injection is 25 to 50 μg, up to 300 μg.

In the group of LIA, epinephrine (0.5 mg), 30 mg of ketorolac acid and ropivacaine (300 mg) were infiltrated into the periarticular soft tissues via surgeons in the operation process. After cutting the bone and before prosthesis implantation, all the tissues injured during the operation were systematically injected by injecting 40 to 50 mL into the collateral ligament and posterior capsule. After implantation of the prosthesis, an injection of 50 to 70 mL through a capsule incision was performed. Ultimately, 50 mL of the ropivacaine (100 mg) without ketorolac or epinephrine infiltrated subcutaneous tissue before the skin is closed.

### 2.4 Outcomes

The main outcome variables were the consumption amount of opioids in the patient-controlled analgesia administered every 6-hour within 24 hours after the operation, and the degree of pain assessed postoperatively via the visual analog scores^[14]^ every 6 hours until 24 hours. Visual analog scores for the pain is between 0 mm (representing no pain) and 100 mm (representing extreme pain). The secondary outcome variables were the side effects associated with opioid, the length of hospital stay, motion range, and the loss of blood collected by the closed suction drainage. The side effects of morphine included nausea and vomiting, retention of urinary, itching and the respiratory depression.

### 2.5 Statistical analysis

All the required analyses were carried out via applying the SPSS for Windows Version 19.0. All data were expressed with appropriate characteristics, for instance, mean, median, standard deviation as well as percentage. The comparison between the 2 groups was conducted through using Mann-Whitney *U* test or the independent samples *t* test. And the comparison of categorical variables between the groups was implemented via the Chi-square test. A value of *P* < .05 was considered as the significant in statistics.

## 3 Results

The clinical outcome variables between groups were shown in Table 1.

## 4 Discussion

Many end-stage knee osteoarthritis patients have improved the mobility, life quality, and improved the pain after TKR.^[15]^ Despite the superb results achieved by TKR, offering an adequate postoperative pain control and rehabilitation is a challenge for the providers, as good management of pain can improve patient outcomes. The lack of a definitive “gold standard” and various programs of pain management after operation indicate that there is much room for improving the standards of care.^[16]^ LIA is effective in the knee surgery. It is on the basis of the systematic infiltration of a epinephrine, nonsteroidal antiinflammatory drugs and long-acting local anesthetics mixture into the tissues around surgical area in order to obtain satisfactory control of pain with slight physiological interference,^[17]^ but the outcomes are not generally optimistic.^[18]^ Epidural analgesia has become a commonly used treatment option, which offers excellent pain relief, but there are also some risks, for instance, epidural hematoma, bradycardia, hypotension, and prolonged lower extremity motor block time and other side effects.^[19]^ Based on the above discussion, we perform this protocol to compare the effectiveness of LIA and intrathecal morphine in the treatment of the postoperative pain after TKR.

## 5 Conclusion

This protocol will provide the evidence on which technique can achieve better analgesia after TKR.

## Author contributions

Lifeng Ma and Ai Guo plan the study design. Bo Yang reviewed the protocol. Jingxin Zhang will collect data. Zhengrong Qi and Zhiyao Li write the manuscript. All authors approve the submission.

**Conceptualization:** Lifeng Ma.

**Formal analysis:** Ai Guo.

**Funding acquisition:** Zhiyao Li.

**Methodology:** Bo Yang, Jingxin Zhang.

**Software:** Bo Yang.

**Writing – original draft:** Zhengrong Qi.
